# Development of a novel single channel arteriole microphysiological system for characterizing leukocyte-endothelial interactions

**DOI:** 10.1017/cts.2025.49

**Published:** 2025-04-04

**Authors:** Sebastian Piombo, Christopher J. Hatch, Chauncey G. Evangelista, Shlomit Radom-Aizik, Dan M. Cooper, Christopher C.W. Hughes

**Affiliations:** 1 Department of Biomedical Engineering, University of California, Irvine, CA, USA; 2 Department of Molecular Biology and Biochemistry, University of California, Irvine, CA, USA; 3 Pediatric Exercise and Genomics Research Center, Department of Pediatrics, School of Medicine, University of California, Irvine, CA, USA; 4 University of California Irvine, Institute for Clinical Translational Science (ICTS), Irvine, CA, USA

**Keywords:** Atherosclerosis, microphysiological systems, device design, inflammatory disease, pediatric origins

## Abstract

**Background:**

Here we present a novel approach to evaluate peripheral blood mononuclear cell vascular adhesion using a microfluidic model designed to approximate the complexity of a human arteriole. While EC monolayer assays are commonly used to investigate leukocyte-EC interactions, we hypothesized that our single channel arteriole (SCA) on a chip would recapitulate the microvasculature more accurately and provide additional insight into the initial stages of atherogenesis.

**Methods:**

This model is comprised of stromal cells embedded in a hydrogel surrounding a channel lined by endothelial cells (EC) that has an inner diameter approximating a small arteriole. Under physiologic shear conditions, the EC take on a phenotype distinct from monolayer cultures, including alignment with the direction of flow.

**Results:**

Significant differences were found between the SCA and monolayer cultures in the expression of key EC and stromal cell markers, including ICAM-1, VCAM-1, PDGFB, aSMA, and KLF2. Indeed, flow-induced PDGFB expression likely mediated the recruitment and differentiation of αSMA-positive cells to the vessel wall. Importantly, the vessels were responsive to stimulation by inflammatory mediators, showing both increased leukocyte adhesion and increased permeability. Finally, mechanically mediated protrusion of the vessel wall into the lumen disrupted flow, producing increased shear over the vessel wall.

**Conclusion:**

In summary, our studies demonstrate the utility of the SCA model for studies of small vessel physiology under both normal and disrupted flow and to lay the groundwork for further development into a model for atherosclerosis. Additionally, our data emphasize the advantages of complex 3D assays over more traditional 2D cultures.

## Introduction

We present here a novel single channel arteriole (SCA) microphysiological system for characterizing leukocyte-endothelial interactions that is capable of examining circulating immune cells obtained from a variety of sources including human donors. Atherosclerotic vascular disease, which has its origins in childhood, remains a key cause of morbidity and mortality across the globe [1,2]. It is now clear that the disease involves a process of chronic inflammation in which diet and lifestyle factors contribute to a cascading pathologic interaction of circulating immune cells and endothelial components of the vasculature [3]. The mechanisms linking circulating leukocytes – known to play a substantial role in the formation of atherosclerotic plaques – with endothelial cells are emerging as a research direction with potential clinical translation for both prevention and treatment [4].

A major challenge in these efforts has been to develop experimental devices and platforms that more closely mimic atherosclerotic plaque formation in humans. Factors such as shear, vascular anatomy, and circulating inflammatory mediators can impact leukocyte adhesion to the vascular wall or aggregation to a nascent plaque. Such processes can interact and influence the formation and/or prevention of vascular injury and plaques. The underlying mechanisms are challenging to examine in vitro using monolayer endothelial cell culture plates [5], an approach that has provided insight into the adhesion of leukocytes to vascular surfaces [6]. Additionally, monolayer models are lacking in much of the complexity that develops in three-dimensional systems, leading to significant differences in endothelial gene expression and function [7]. The SCA platform described here begins to address these challenges and is composed of human endothelial cells (EC) overlaying a stromal cell-rich matrix, closely modeling the complex composition of naturally occurring tissues [8]. Using this model, we began to address the potential effectiveness of the SCA as a platform to examine key elements of the role of leukocyte-endothelial interaction in atherosclerotic vascular disease. Specifically, we tested the effect of physiologic flow and shear conditions on recruitment of stromal cells to the EC layer and induction in these cells of smooth muscle alpha-actin (αSMA); induction of shear-responsive genes; and quantification of leukocyte-endothelial adhesion after treatment with specific inflammatory mediators known to influence leukocyte transmigration, namely ICAM-1 and VCAM-1. We compared observations made from the SCA with the monolayer approach.

## Materials and methods

### SCA platform design and fabrication

**Figure 1.**
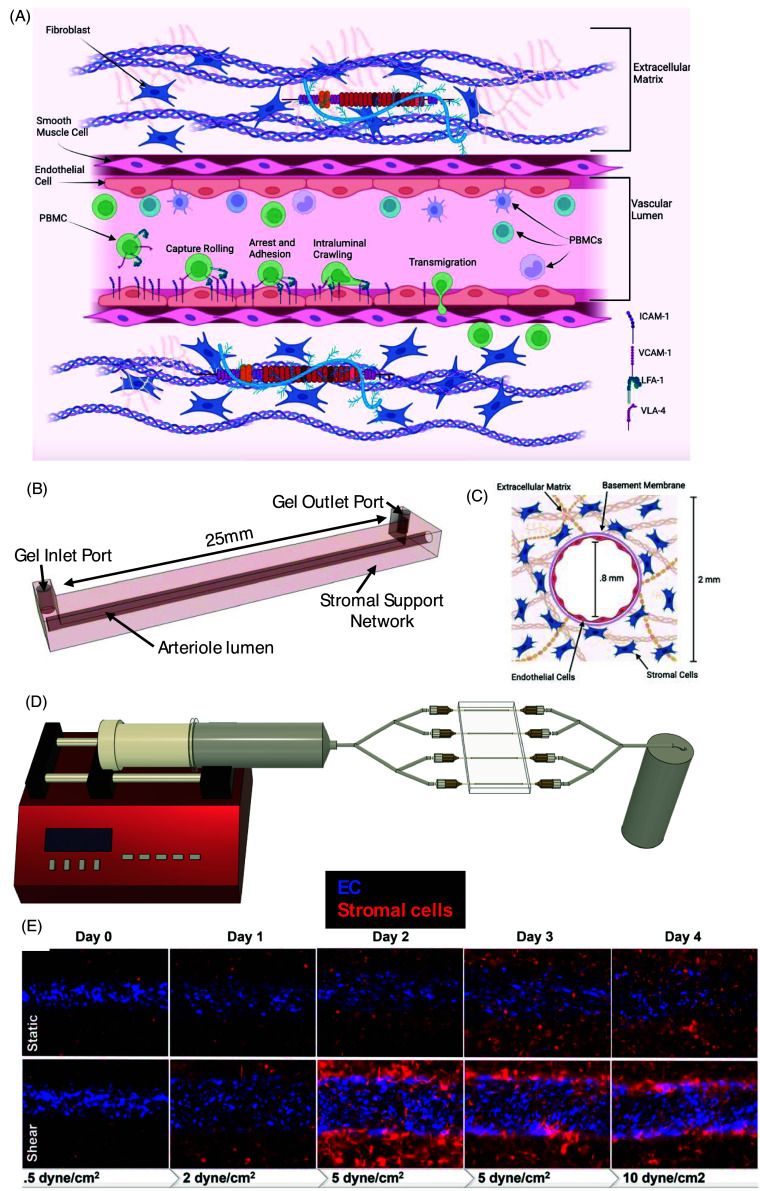
Single channel arteriole chip design and developmental timeline under static and shear conditions. (A) A schematic of the arterial vessel showing EC, stromal cells, SMC, extracellular matrix, and EC adhesion molecules relevant to leukocyte extravasation. (B) Tissue chamber schema with relevant measurements. (C) Cross-section schematic of SCA showing cell and gel arrangement. (D) The SCA device arranged with media inlets and outlets in parallel, driven by a programmable pump. (E) SCA development over the course of four days under static and increasing shear conditions.

Using a laser-cut acrylic mold, the base of the chip is principally based on a two-part polyurethane liquid plastic that acts as a master mold for subsequent polydimethylsiloxane (PDMS) replicas. The base layer of the chip is fabricated from PDMS, which following an overnight curing has inlet and outlet holes punched prior to being mounted onto a 50 –60 mm, 0.5 mm thickness glass slide. The slide and PDMS chip are bonded by exposure to oxygen plasma for three minutes and again allowed to set overnight in a 60°C oven. The bonded chip then has male 1/16” bore Luer lock valves (McMaster-Carr, 75165A675) inserted and affixed into place using PDMS at either end of the tissue chamber. Following the insertion of the Luer lock inlets a 0.8 mm diameter nitinol wire (McMaster-Carr, 8320K34) is inserted through the apertures to bridge the tissue chamber and provide the space that will eventually form the lumen of the arteriole (Figure 1A–C). This final assembly is then sterilized by exposure to UV light for 30 minutes and is viable for use for up to 4 months prior to plastic degradation.

### Cell culture

There were two primary cell types used in the experiments described. Human umbilical vein endothelial cells (HUVEC, referred to as EC) were isolated from fresh cord tissue acquired through the UCI Douglas Hospital with internal approval as previously described [9,10]. Although HUVEC occupy a vein position – carrying blood toward the (fetal) heart – they are actually carrying oxygenated blood from the placenta, and thus have some arterial characteristics. EC were cultured on gelatin-coated cell culture flasks in EGM-2 (Lonza, CC-3162) medium. Normal human lung fibroblasts (NHLF, Lonza) were cultured in DMEM (Corning, 10027CV) with 5% FBS (VWR, S1700-100). Both HUVEC and NHLF were transduced with lentiviruses encoding fluorescent proteins (mCherry/Addgene, 36084 or Azurite/Addgene, 36086) and were used between passages 5–9. All cells were cultured at 37^o^ C/20% O_2_/5% CO_2_ in incubators with cupric sulfate baths in the base.

### SCA platform loading

Immediately following UV exposure, the chip was ready for loading. A solution of fibrinogen (Sigma-Aldrich, 9001-32-5) was prepared at 12 mg/mL in EGM-2 and kept at room temperature while the NHLF were lifted from their flasks and suspended in the gel-cell mix at a concentration of 2 million cells/mL. This solution was rapidly mixed with thrombin and pushed through the negative space within the chamber of the chip. This was repeated in quick succession through the next three devices on the chip before the devices were placed in an incubator for 30 minutes to allow for gel polymerization. Following polymerization the nitinol wires were removed from the matrix, leaving a vacant channel. Each channel was then flushed with laminin (0.5 mg/mL, Life Technologies) to support the adhesion of EC and provide the basis of a basement membrane. The EC suspension was prepared in EGM-2 at a concentration of 4 million cells/mL and perfused through the lumen prior to being capped at either end and allowed to rest for an hour. This allows for the EC to settle and anchor on the matrix. After an hour the devices were flipped to ensure even distribution of the cells.

### Platform flow metrics

Following an overnight culture post-loading, the devices were hooked together in series or in parallel using 1/16” polyurethane microfluidic tubing (Cole-Parmer, EW-21942-76) segments. Once assembled, a syringe pump (New Era Syringe Pump Systems Inc.) was hooked onto the top of the chip and set to flow at a rate of 2 mL/hour, slowly pushing the EC that did not attach to the matrix to be removed from the lumen of the devices. After five hours of this flushing, the flow rate across the arterioles was increased to a rate of 5 mL/hour for 24 hours. During this period, the gel continued to set as the NHLF remodeled and deposited additional ECM in the fibrin gel, allowing for the devices to accommodate higher flow. After 24 hours of flow, the media was replaced with a blood substitute media consisting of EGM-2 mixed with xanthan gum (Sigma, 11138-66-2) to achieve a viscosity of 3.5 cP. This viscous blood substitute is then placed into the reservoirs of the New Era Syringe pump system and the flow was set to 25 mL/hour for 48 hours. Following this 48-hour period of flow with the blood substitute, the flow rate was set to 50 mL/hour to achieve a shear stress of 10 dyne/cm^2^ across the arteriole wall. This rate can be maintained for up to several days with the devices remaining functional. For all experiments requiring an inflammatory stimulus, the SCA devices or monolayer culture were perfused with TNF-a (Sigma, 11088939001) at a concentration of 9 ng/mL for 24 hours.

### Peripheral blood mononuclear cell (pbmc) perfusion

The PBMCs were isolated from donated blood provided by the UCI Institute for Clinical and Translational Science blood donor system using Ficoll gel gradient CPT tube (BD Biosciences, 362753). These isolated PBMCs were then rinsed and brought to a concentration of 1.5 million cells/mL before being stained with CellTracker Green dye (Thermo Fisher, C2925) according to the manufacturer’s instructions. Post-staining the PBMCs were assessed for viability with trypan blue, counted, and rinsed. Following perfusion at a shear stress of 10 dyne/cm^2^ for 24 hours, the devices were removed from the incubator and fresh blood replacement media with CellTracker-labeled PBMCs at a concentration of 250,000 cells/mL were added. This cell suspension was then perfused across the single channel arteriole chip for 24 hours and imaged at the 6-, 12-, and 24-hour timepoints. Prior to imaging, the devices as well as the monolayer comparisons were gently washed with media to remove any non-adherent cells from the system.

### Imaging

At the 6-, 12-, and 24-hour time points, the channels were imaged along the entirety of the arteriole length using a Nikon Ti-E Eclipse epifluorescent microscope with a 4xPlan Apochromat Lambda objective. These images were then analyzed using FIJI [10] and adherent and extravasated labeled PBMCs were enumerated. Once cell counts had been established final leukocyte adhesion was normalized to endothelial surface area of the channel.

### RNA isolation and qRT-PCR analysis

In order to extract RNA from the tissue chamber, the glass coverslip was gently pared off the PDMS mold and both the surface of the slip and the tissue chamber were treated with 200 uL of Trizol and the resulting cell lysis volume was collected and prepped using a Quick RNA micro prep kit (Zymo Research, R1051) according to manufacturer’s instructions. Once the RNA isolation was complete, a cDNA library was prepared using Zymo-Seq Universal cDNA kit (Zymo Research, R3001) and qRT-PCR was performed for the gene targets in triplicate. The housekeeping gene selected to compare the gene targets was 18s ribosomal RNA. Monolayer HUVEC and SCA samples were standardized against monolayer HUVEC as well as CD31 expression, which does not change under flow (unpublished observations). Primer sequences were designed and ordered via PrimerQuest Tool and synthesized using IDT technologies.

### SCA permeability assay

Once the SCA was fully developed by day 4, a 70 kDa FITC-dextran (Sigma-Aldrich, D1823) was diluted in blood substitute media and flowed through the devices at a rate of 10 mL/hour. Images were taken at 5-minute intervals, and mean fluorescence intensity was quantified in a demarcated region immediately adjacent to the lumen wall. Control devices were developed under standard blood substitute media conditions, and then, TNF-a was added at 9 ng/mL for 24 hours prior to the permeability assay.

### Impingement of SCA

Prior to the fabrication of the platform, 1.5 mm biopsy punches were placed on opposite sides of the channel. Following the loading of the devices and development of the vessels over the course of four days, 1/8” diameter polypropylene spheres (McMaster-Carr, 1974K2) were injected into the biopsy punch space, displacing the outer wall of the PDMS channel into the stromal compartment. This displacement in turn created a consistent occlusion across the lumen of the arteriole of up to 50%, dependent on the placement of the biopsy punch during fabrication. Several of these occlusions can be placed along the length of a single arteriole, enabling complex flow dynamics to arise in an otherwise straight channel. These occlusions were stable fixtures along the length of the channel and remained in place for up to three days after introduction.

### Computational fluid dynamics

Images of the SCA were imported into AutoCAD as a PDF underlay. The boundaries were manually traced. The traced vessels were imported into COMSOL 5.2.1. The velocity and shear stress were calculated using the laminar flow steady-state model, with blood as the fluid material for the normal SCA, whereas the RANS Algebraic yPlus turbulence model was used for the impinged devices. The inlet velocity was set based on the steady flow rate driven by the linear syringe pump.

### Plotting and statistical analysis

All plots were generated using either Microsoft Excel or ggplot2 [12] in R, and figures were generated using Bio Render. One-way ANOVA was used for qPCR analysis (i.e. expression of PDGFB, aSMA, KLF2, ICAM-1, and VCAM-1) with a Tukey Test or Dunnett test (VCAM-1 only) to determine significances between groups. Comparison between TNF-a and control SCA for both the permeability assay was made using an one-way repeated measures ANOVA with a Bonferroni correction. The criterion for statistical significance was alpha less than 0.05 for all tests.

## Results

### Establishment of the SCA

The vessel wall is complex, being composed of EC, perivascular cells, stromal cells, and extracellular matrix/basement membrane (Figure 1A). We generated a single channel with inputs at each end, surrounded by gel (Figure 1B, C). Once the gel containing the stromal cells sets, the wire is carefully removed and the resulting 800 μm channel is seeded with EC (Figure 1C). The EC became confluent over the next 2–3 days as the shear slowly increased, using a programmable pump (Figure 1D), to 10 dynes/cm^2^. During this time, the EC aligned with flow and the stromal cells begian to undergo distinct morphological changes (Figure 1E). Notably, as the cells proliferated and remodeled the matrix they were recruited to the basolateral side of the EC lining the lumen of the SCA, where they wrapped the vessel in a manner reminiscent of pericytes and smooth muscle cells. If high flow rates were established immediately after seeding, the EC were rapidly stripped from the walls and the gel collapsed, indicating that the remodeling that occurred generated a stronger vessel more capable of sustaining high shear.

### Gene expression differences between monolayer and SCA

**Figure 2.**
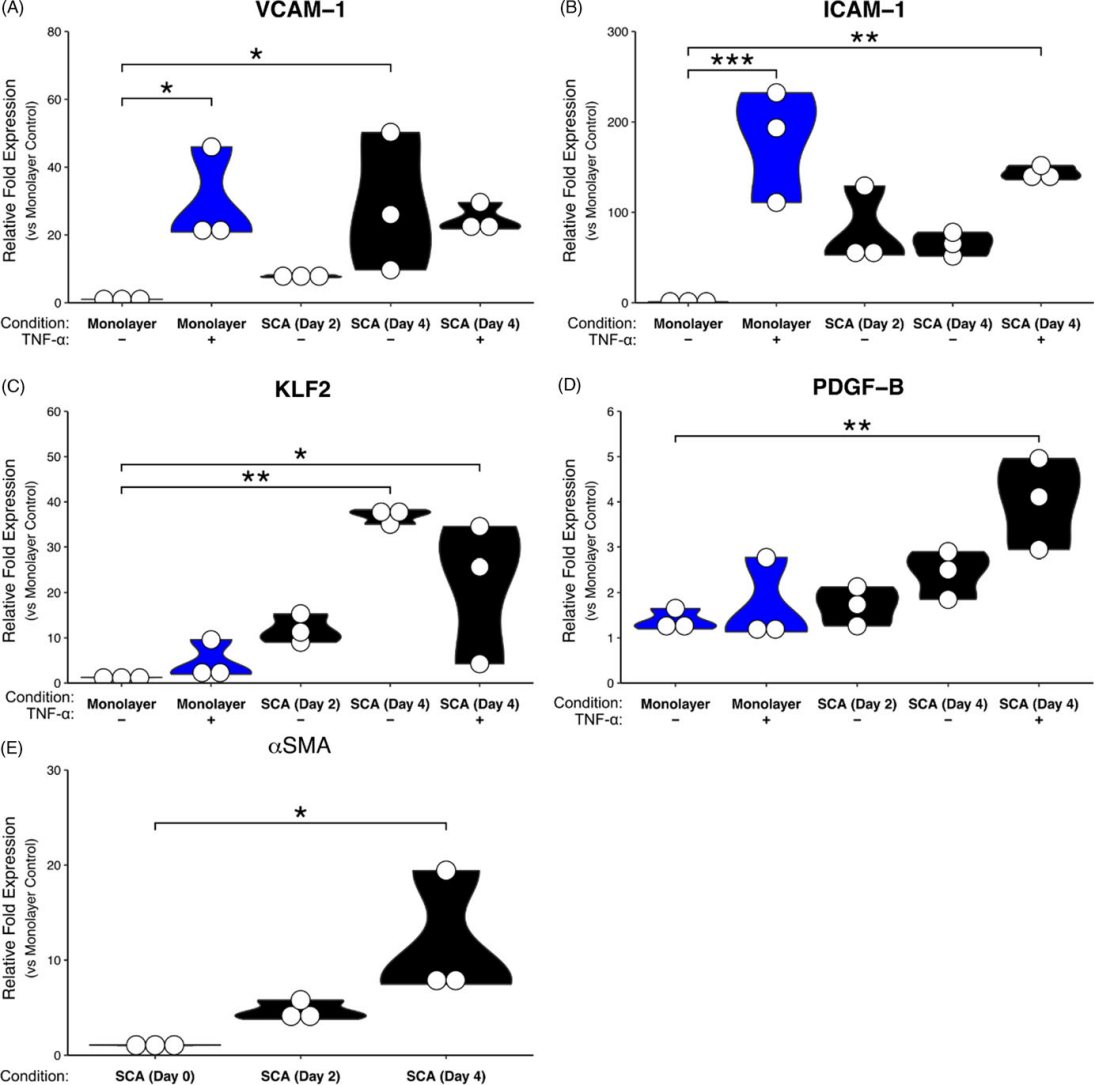
Gene expression analysis of developmental and inflammatory markers in SCA and EC monolayer. qPCR analysis of (A) ICAM-1, (B) VCAM-1, (C) KLF-2, (D) PDGFB, and (E) αSMA expression in both EC monolayer and SCA over time. Fold change normalized to HUVEC monolayer. * *p* < 0.05; ** *p* < 0.01, *** *p* < 0.001.

Following four days of development, RNA was harvested from the tissue chamber and qPCR was performed. EC monolayer cDNA libraries were generated from the same cell preparation used to load the SCA. Consistent with the establishment of physiologic flow, we saw upregulation of KLF2, the major mediator of flow-generated signals in EC, as well as induction of PDGFB, which is necessary for recruitment of perivascular cells such as pericytes and SMC (Figure 2C–E). We saw further upregulation of PDGFB and KLF2 expression as the SCA tissue matured over the course of 4 days (Figure 2 C, D). The recruitment of stromal cells to the vessel wall correlated with an increase in αSMA expression, indicative of fibroblast differentiation to smooth muscle cells (Figure 2E). We also found significant differences in expression of the EC leukocyte adhesion molecule genes ICAM-1 and VCAM-1 when comparing monolayer to SCA (Figure 2A, B). Interestingly, baseline levels of both genes were much lower in monolayer compared to SCA and the fold increase in response to TNF-α was correspondingly higher. A static monolayer is not a physiologic model, although it has been extensively used for study of adhesion molecule expression and function. We believe that the more modest changes seen in the SCA, with a higher basal level of expression, likely reflect the response of EC to flow conditions in this physiologic model. Whether the expression at basal levels is mediated (indirectly) by the flow sensor KLF2 can be resolved in future studies.

### PBMC adhesion and extravasation

**Figure 3.**
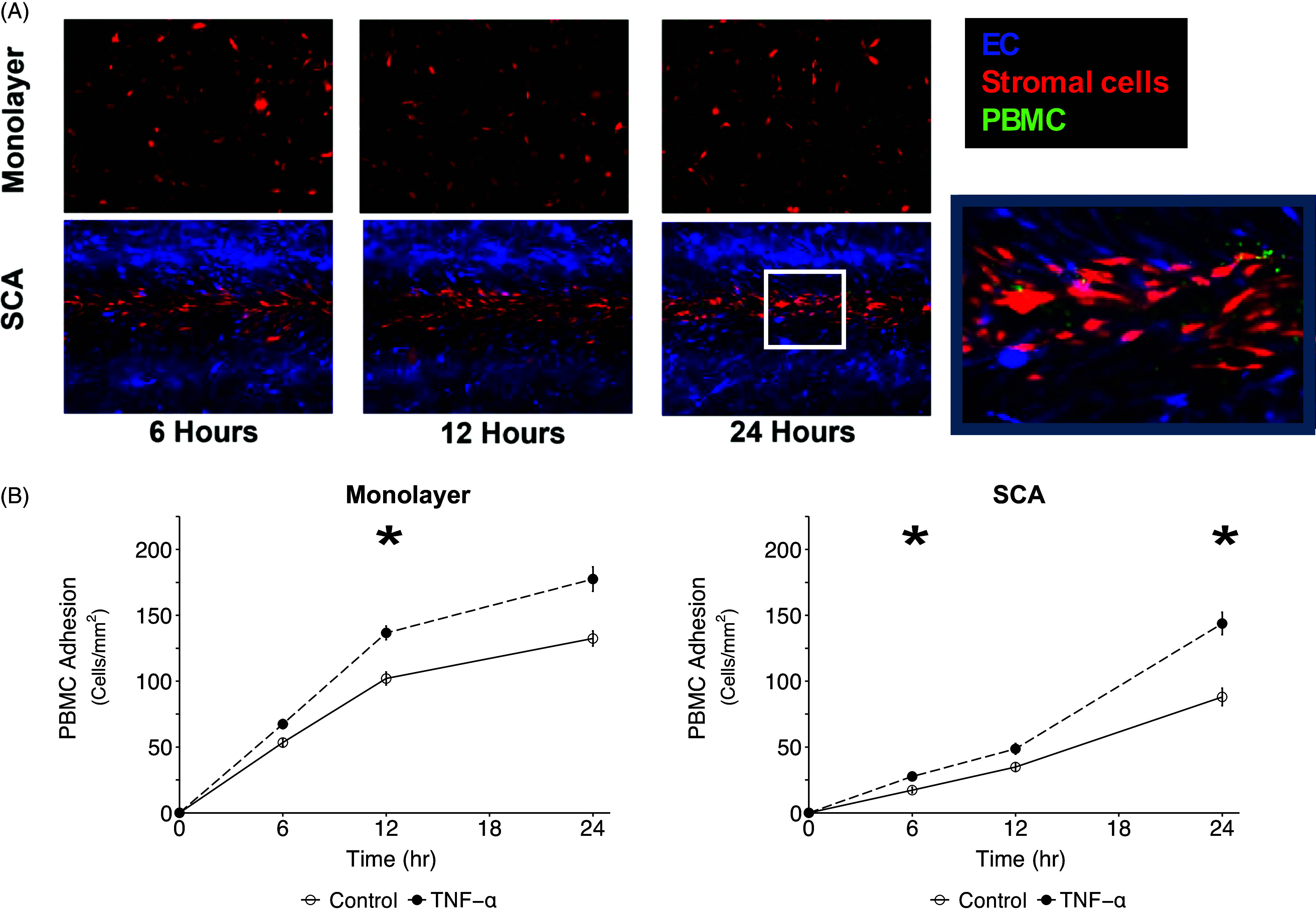
PBMC adhesion in EC monolayer and SCA is augmented by an exogenous inflammatory stimulus. (A) Fluorescent imaging of PBMCs adhering to both EC monolayer and SCA over 24 hours with GFP labeled PBMCs visualized adherent to mCherry endothelial cells. (B) Quantification of PBMC adherence to monolayer and SCA vessel walls in the presence or absence of TNF-a stimulation. * *p* < 0.05.

Leukocyte extravasation during inflammation is strongly dependent on expression of ICAM-1 and VCAM-1, and we found both to be inducible by the inflammatory mediator TNF-α, although induction was less dramatic under flow conditions than is seen in monolayer (Figure 2) [13]. We next examined the ability of the SCA platform to model leukocyte adhesion and extravasation under flow. Once devices were mature (after 4 days), PBMCs were perfused through the vessel, and adhesion and extravasation were compared to monolayer cultures (Figure 3A). Adhesion and extravasation of the PBMCs were standardized to the surface area of EC in each model. In both cases, we observed a time-dependent increase in PBMC adhesion, and this was significantly increased by prior treatment of the cells with TNF-α (Figure 3B). We found more rapid adhesion to the monolayer and a higher number of cells per area, which is consistent with the less demanding environment in the monolayer cultures where flow is absent. Despite the large fold increase in ICAM-1 and VCAM-1 expression in monolayer in response to TNF-α (Figure 2), we did not see a corresponding increase in PBMC adhesion.

### Permeability of SCA vessel in response to TNF-a

**Figure 4.**
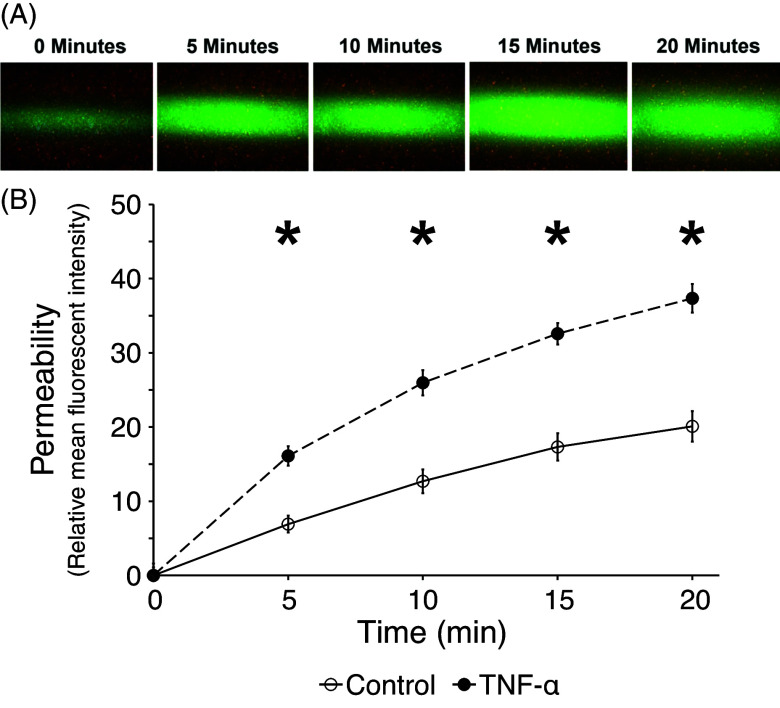
TNF-a induces vascular leak in SCA under flow conditions. (A) Fluorescent imaging over time of SCA with 70 kDa FITC-dextran perfusing through the system. (B) Mean fluorescence intensity adjacent to vessel wall over 20 minutes. * *p* < 0.05.

A key change that vessels undergo under inflammatory conditions is a weakening of junctional contacts leading to an increase in vessel wall permeability. To determine whether the SCA model could recapitulate this phenomenon we treated vessels with TNF-α or control and then assessed changes in permeability over time to 70kDa-FITC dextran, which models serum albumin (68kDa) in size (Figure 4A). Over the course of 20 minutes, there was a slow leak of FITC-dextran from the control vessels (Figure 4B), consistent with the usual bulk flow of fluid into peripheral tissues. This leak was significantly enhanced by pretreatment of the SCA with TNF-a (Figure 4B), again consistent with the known role of TNF-a in vascular inflammation.

### Impingement of SCA and downstream flow perturbation

**Figure 5.**
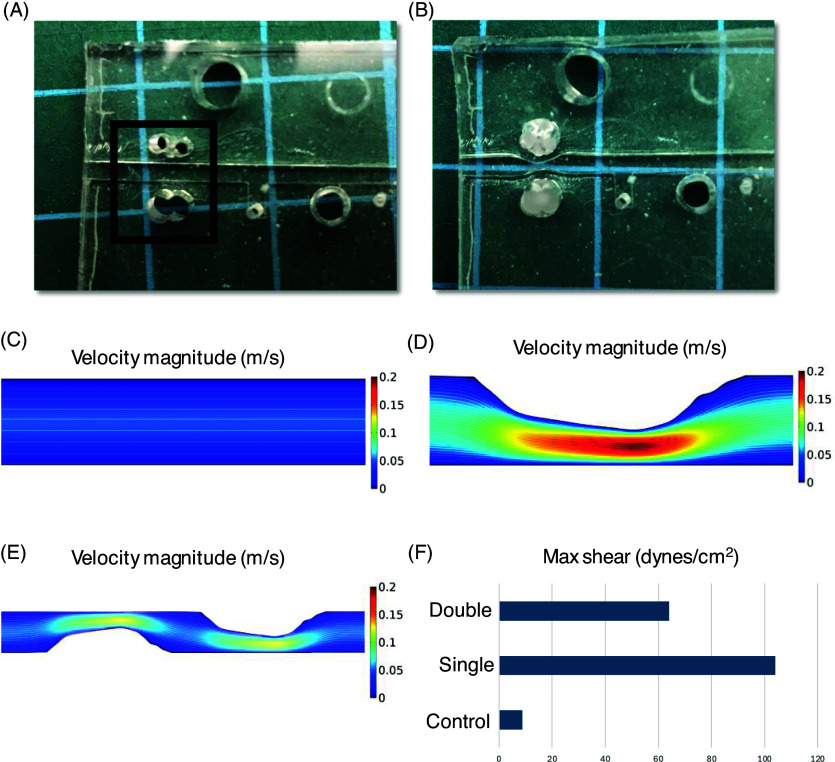
Fabrication and flow dynamics of impinged SCA. (A) Impingement fabrication of SCA devices. Prior to loading of gel-cell mixture holes are punched. (B) Following cell/gel loading polypropylene beads are injected proximal to the tissue chamber displacing the channel wall. (C) Smooth velocity gradient in control SCA. (D) Disturbed and accelerated flow through a singly impinged SCA. (E) Disturbed and accelerated flow through a doubly impinged SCA. (F) Dramatically increased vessel wall shear in impinged SCA vessels.

To better understand the progression of atherosclerosis, particularly the latter stages where plaque impingement into the lumen generates disturbed flow patterns, we generated a proof-of-concept modification to the SCA model (Figure 5). One or two beads were inserted into preformed holes in the gel adjacent to the vessel, which had the effect of reproducibly deforming the extracellular matrix such that the vessel wall impinged on the lumen (Figure 5A, B), in much the same way as an atherosclerotic plaque. The impingements introduced into the channel can occupy up to 50% of the vessel lumen, accurately recapitulating the effects of mild to moderate size atherosclerotic plaques. These constrictions of the channel lead to changes in the fluid flow pattern, as assessed by COMSOL MultiPhysics modeling (Figure 5C–F). In control vessels, as expected, we could calculate high levels of shear immediately adjacent to the vessel wall, and this fell off smoothly toward the center of the lumen where flow is highest (Figure 5C). In contrast, where the lumen is impinged by outside pressure from the bead we saw dramatically disturbed flow, with an almost 5-fold increase in shear stress on the vessel wall at the site of these artificial lesions (Figure 5D, E, F). We did not detect turbulence, likely due to the Reynolds number being too low at these flow velocities. We are currently examining ways to introduce a bifurcation or other flow-splitter that might generate turbulent flow. Nonetheless, the current model does demonstrate accelerated flow and shear over the surface of the artificial “plaque,” which could be used to model how shear may interact with damaged and inflamed endothelium, a forerunner to plaque rupture.

## Discussion

To accurately evaluate leukocyte-endothelium interactions, it is critical to recapitulate the *in vivo* factors that contribute to adhesion and transmigration across the endothelial barrier [14]. Our model provides a promising, novel addition to current human EC cultures, significantly enhancing mechanisms elucidated in monolayer cultures. By incorporating microfluidic engineering, we have developed a readily replicable, three-dimensional arteriole organoid that operates within the normal physiological bounds of an *in vivo* arteriole with regard to shear stress and lumen size [15]. Additionally, incorporating stromal cells/fibroblasts in a complex extracellular matrix further improves upon previous models such as Transwell assays where there is mechanical separation of the two distinct cell lines [16]. In our SCA, fibroblasts are recruited to the developing vessel wall where they differentiate into αSMA-expressing SMC. The SCA is generated within a relatively short time frame as the combination of cells present in the tissue chamber rapidly adapts to the high shear force present to produce a confluent EC-lined lumen supported by SMC and stromal fibroblasts.

Our SCA enhances previous observations that have showed extravasation of monocytes was highly regulated by the structural integrity of the endothelial monolayer using a microfluidic device [17]. Building upon prior work in this field, the device presented here has several unique components. The ability to introduce impingements into the SCA is a novel strength and allows for the study of flow perturbation in response to plaques of a variety of sizes, as well as studies on subsequent leukocyte-endothelial interactions downstream of the impingement. Additionally, our device is highly scalable compared to other model systems, accommodating up to a dozen channels operating in parallel and connected via a single pump system, allowing for high throughput studies to be performed. Our SCA allowed us to extend these observations by exploring biomarker, inflammatory mediator, gene expression, and anatomic disruption on key features of the leukocyte-vascular interaction using immune cells derived from human subjects. Thus, the stage is set for studies using leukocytes and leukocyte subtypes obtained from human participants with specific characteristics such as adults with known atherosclerosis, or children at risk for the development of vascular disease due to obesity or familial hyperlipidemia. The SCA model could then be employed to gauge the effect of potential drug or lifestyle interventions on the mechanisms that contribute to the endothelial-circulating immune cell interaction.

Upon generation of the fully matured arteriole in the chamber, there were distinct morphological and gene expression pattern differences between the SCA and the same cells in 2D culture, further demonstrating the value of approximating the complexity of native tissue in the human body. The adhesion and extravasation of leukocytes across the endothelium is a multifactorial process dependent on the expression of adhesion markers, the number of collisions between the two cells, and the priming effect of shear stress on both immune cells and vasculature [5]. The SCA can capture these complexities in a controlled microfluidics-based platform. The marked differences between the SCA and 2D assays allow for real-time measurements of the functional phenotype of PBMCs that more accurately model the dynamics of leukocytes in the human body.

CVD and atherogenesis are processes that are highly dependent on shear stress and vessel morphology, and the SCA provides an ideal basis to evaluate the initial disease steps that have proved to be elusive to study. We have shown a proof-of-concept that the vessel wall can be impinged leading to dramatic changes in flow and shear at the endothelial luminal surface. We envisage future studies incorporating macrophages and a high lipid load to further develop the SCA as a model for atherosclerosis. Additionally, we are interested in continuing to pioneer means by which to exacerbate turbulent flow through the devices by introducing upstream bifurcation points or increasing flow across the devices once the devices are fully developed following day 4. In addition, monocyte/macrophages can be added either directly to the tissue as it is constructed or delivered through the vessel once it has formed, the latter being a more physiologic route. The framework for these devices holds great promise for furthering our collective knowledge of atherogenesis as well as other leukocyte-endothelial pathologies.
